# Disentangling the Relationship Between Social Protection and Social Cohesion: Introduction to the Special Issue

**DOI:** 10.1057/s41287-022-00532-2

**Published:** 2022-05-02

**Authors:** Francesco Burchi, Markus Loewe, Daniele Malerba, Julia Leininger

**Affiliations:** 1 Deutsches Institut für Entwicklungspolitik/German Development Institute (DIE), Research Programme “Transformation of Economic and Social Systems”, Tulpenfeld 6, 53113 Bonn, Germany; 2 Deutsches Institut für Entwicklungspolitik/German Development Institute (DIE), Research Programme “Transformation of Political (Dis-)order”, Tulpenfeld 6, 53113 Bonn, Germany

**Keywords:** Social protection, Social cohesion, Cash transfers, Public works, Graduation programmes, Empirical evidence, Mixed methods, D63, H41, H53, H55, I38

## Abstract

While there is substantial evidence of the effect of social protection on poverty and vulnerability, limited research has focused on societal outcomes. This paper serves as introduction to a special issue (SI) examining the relationship between social protection and social cohesion in low- and middle-income countries. Over the last years, social cohesion has emerged as a central goal of development policy. The introduction and the papers in the SI use a common definition of social cohesion as a multi-faceted phenomenon, comprising three attributes: cooperation, trust and inclusive identity. This introductory article provides a conceptual framework linking social protection to social cohesion, shows the current empirical evidence for the bi-directional linkages, and highlights how the papers in the SI contribute to filling existing research gaps. In addition to this introduction, the SI encompasses seven papers, covering different world regions and social protection schemes, and using different quantitative and qualitative methods.

## Résumé

Il existe d’importantes données probantes concernant l'effet de la protection sociale sur la pauvreté et la vulnérabilité. Cependant, les études qui se penchent sur les effets au niveau de la société sont peu nombreuses. Cet article fait office d'introduction à un numéro spécial qui étudie la relation entre la protection sociale et la cohésion sociale dans les pays à revenu faible et intermédiaire. Au cours des dernières années, la cohésion sociale est devenue un objectif central de la politique de développement. L'introduction et les articles de ce numéro spécial utilisent une définition commune de la cohésion sociale en tant que phénomène à multiples facettes, comprenant trois attributs: la coopération, la confiance et l'identité inclusive. Cet article introductif fournit un cadre conceptuel reliant la protection sociale à la cohésion sociale, montre les preuves empiriques actuelles des liens bidirectionnels et met en lumière la façon dont les articles de ce numéro spécial contribuent à combler les lacunes de la recherche existante. En plus de cette introduction, le numéro spécial comprend sept articles qui couvrent différentes régions du monde et divers régimes de protection sociale, et font usage de différentes méthodes quantitatives et qualitatives.

## Setting the Scene

The development community has shown an increasing interest in social protection since the start of the new millennium. This trend is due to the increasing evidence that sustainable poverty reduction is difficult to achieve without investment in social protection because economic growth does not usually trickle down to reach an entire population. Moreover, social protection is increasingly recognised as a key driver of economic growth, especially if it incentivises productive investments by low-income households. And, finally, an increasing number of publications (e.g. Babajanian 2012; Evans et al. 2019; Loewe et al. 2020; Molyneux et al. 2016) show that social protection can also contribute to political and broader societal developments, even if such effects are often not its primary intended goals. This special issue contributes to this wide-ranging and multi-faceted debate, focusing specifically on social cohesion.

Over recent years, social cohesion has emerged as a central goal of development policy, as demonstrated by numerous publications by international organisations and bilateral donors such as UNDP (2016), the World Bank (Marc et al. 2012), and the OECD (2011). The reasons for this are three-fold. First, societies that are more cohesive are believed to be more resilient, in particular with respect to natural disasters and public health crises such as the ongoing global Covid-19 pandemic (Abrams et al. 2020; Townshend et al. 2015). Second, social cohesion fosters societal peace (Fearon et al. 2009; Gilligan 2014; UNDP 2020). Third, social cohesion contributes to local community development (e.g. Wilkinson et al. 2017), which often depends on a community’s ability to agree on common goods to be created for the benefit of all community members.

Identifying policies that foster social cohesion is therefore crucial, not least because political and social polarisation is currently rising in many countries worldwide (Carothers and O’Donohue 2019). Social protection is potentially one of these policies. Examining its effects on social cohesion is particularly important in the context of the Covid-19 pandemic’s impact on health, societies and economic development globally. People around the world feel more vulnerable, which can undermine resilience and thereby bring about societal and political instability.

However, the relationship between social protection and social cohesion is unlikely to be a one-way street: socially cohesive societies are deemed, in their turn, to provide better and more all-encompassing and acceptable social protection systems because their members share similar values; a shared understanding of the common good helps to identify generally acceptable compromises for the design of social protection systems.

This bi-directional relationship, even though it is quite intuitive, has received only limited attention so far. One major reason is that there is no universally agreed concept of social cohesion and no established set of indicators to measure it (see Babajanian 2012; Bastagli et al. 2016). In this special issue (SI), we address the problem by relying on a clear definition of social cohesion, which is quite similar to many definitions already suggested by the existing literature but is sufficiently narrow for straightforward operationalisation. According to this definition, social cohesion is composed of three main attributes: cooperation for the common good, trust and inclusive identity (Leininger et al. 2021a).

The SI examines the possible effects of social protection on social cohesion and—though less so—those of social cohesion on social protection. It aims to address three sets of interrelated guiding research questions. The *first set* is whether different social protection schemes generate effects on social cohesion, and which ones have the strongest effects and on whom. Only the direct beneficiaries or the entire population? Mainly people in poverty? Mainly people working in the formal sector? Mainly women or men? The *second set* concerns the conditions under which these effects materialise. When exactly do they arise? In which contexts? Does it matter if a social protection scheme has been set up by the state or by other actors? Does the quality of targeting or quality of benefit delivery play a role? How important is the reliability and institutional durability of the schemes? And the *third set* of questions is whether social protection influences all aspects of social cohesion in the same way. Or does it perhaps affect mostly inclusive identity because beneficiaries (and possibly others) all feel better integrated into society—or horizontal trust because social benefits can bridge gaps and overcome hostility between different socio-economic classes? Likewise, we have to ask if all of these components are equally important for the existence and functionality of social protection schemes. And at the same time, we ask what role social cohesion plays for the planning, design, setup and operation of social protection programmes.

The remainder of this introductory article is structured as follows. The next two sections present the concepts of social protection and social cohesion endorsed in this SI. The fourth section introduces the conceptual framework linking social protection to social cohesion, while the fifth reviews the existing empirical literature on the causal effects going either way. The last section presents the key findings of the papers in this SI as well as the gaps remaining in research so far.

## Social Protection

The notion of social protection in international development is still quite ambiguous. Most people would probably agree with the definition of social protection as:

… the entirety of policies and programmes that protect people against poverty and risks to their livelihoods and well-being. (Loewe and Schüring 2021, p. 1)

This means that social protection includes all measures that help people in their efforts to (i) *prevent risks* (e.g. by healthy diet, cautious behaviour in street traffic, safety at work, vaccinations, social distancing during pandemics), (ii) *mitigate risks* (e.g. by crop or income diversification, the accumulation of savings or insurance or risk hedging) and (iii) cope with the effects of risks (e.g. by credit or income-support to people in need).

At the same time, there is still disagreement on some feature of social protection, such as the following: (i) *Who can provide social protection* (just the state—or also private actors such as private health or life insurance companies, social welfare organisations, informal self-help groups or society-based mutual support networks) and (ii) *Which risks* should be covered (just health and life-cycle risks such as longevity and work-disability—or also political, macroeconomic, natural and environmental risks such as theft, terrorism, drought, soil degradation or business failure).

At the very minimum, though, social protection includes (i) *non-contributory transfers* (direct and indirect, in cash or kind), (ii) *social insurance* (which is contributory and activated in case of contingencies only), (iii) *micro-insurance*, (iv) *labour market policies* (passive and active) and (v) *social services* (such as therapy, training, or rehabilitation) (Loewe and Schüring 2021).

The core purpose of social protection is to reduce vulnerability and poverty in a country by preventing people from falling into poverty (*preventive function*), providing support to those who are living in poverty (*protective function*) and enabling low-income earners escape from poverty (*promotive function*) (Loewe 2009a). Thereby, it contributes to nutrition, education and health because it allows even low-income people to buy food, consult a physician when they are sick and send their children to school rather than work (Burchi et al. 2018; Kabeer 2014; Strupat 2021). And social protection is also one of the most powerful tools to reduce income inequality between social classes (Inchauste and Lustig 2017) and genders (Holmes and Jones 2013).

However, social protection can also have a *transformational function* by addressing the root causes of poverty and vulnerabilities, such as unequal power relations or unjust distribution of public resources. This function has been less explored so far, given the indirect link between many social protection programmes and transformational outcomes of interest, such as social equity and inclusion, empowerment and rights (especially labour laws).

At the same time, social protection matters also for economic development (Barrientos and Malerba 2020; Loewe 2009a): On the one hand, it enables even low-income households to address risks, smooth income volatility and improve inter-temporal allocation of income. Thereby, it improves the lifetime utility of households and reduces pressure put on networks and society as a whole to provide support for people in need who have failed or omitted to make provision for themselves. On the other hand, social protection encourages low-income earners to make investments and thereby improve their future income expectations. This effect is due to the fact people with low-income and insufficient social protection tend to deposit any possible small savings in a safe place, from which they can easily withdraw the savings without penalty whenever they suffer a loss caused by bad harvest, illness, unemployment or any other risk. This preference changes only once people enjoy reliable and sufficient social protection against at least their most fundamental risks. Some empirical evidence shows that from then on, people start investing at least some of their savings in machines, new modes of production, training or better education for their kids (Gehrke 2019). Investments like these bring about new risks (investment failure) but they raise future income expectancy. Likewise, Borga and d’Ambrosio (2019) find that beneficiaries and non-beneficiaries alike increased their investment in asset formation and livestock holding in response to the launch of cash-for-work programmes in India and Ethiopia. And Bastagli et al. (2016) confirm a clear relationship between cash transfer receipt and increased school attendance, the use of health services and investment in livestock and agricultural assets. If well designed, social protection can thus be a key driver of pro-poor growth: growth that benefits predominantly low-income people (Alderman and Yemtsov 2014; Bhalla et al. 2021; Ravallion et al. 2018; Sabates-Wheeler and Devereux 2011).

## Social Cohesion

Social cohesion refers to the ties or the “glue” that hold societies together (Durkheim 1999). Overall, there is a broad agreement that social cohesion is a complex, multi-faceted phenomenon, encompassing a *horizontal* and a *vertical dimension* (Jenson 2010). While early studies only equated social cohesion with the relationship among individuals and groups in a society (*horizontal dimension*), over the last years, equal emphasis has been placed on the relationship between individuals and state institutions (*vertical dimension*) (Chan et al. 2006; OECD 2011; Langer et al. 2017; Lefko‐Everett 2016).

In the search for an operational definition of social cohesion, we adapt the minimalist approach suggested by Chan et al. (2006), according to whom the concept should be “thin”, including only the core attributes and excluding the determinants (e.g. inequality) and the outcomes (e.g. peace) of social cohesion. As often generally stated in academic and policy debates, inequality is likely to play a key role in determining social cohesion in a society (Leininger et al. 2021a). However, verifying the relationship between social cohesion and inequality analytically is not possible if they are part of the same concept. Against this background, in this SI, we endorse the definition provided by Leininger et al. (2021a):

Social cohesion refers to both the vertical and the horizontal relations among members of society and the state as characterised by a set of attitudes and norms that includes trust, an inclusive identity and cooperation for the common good. (Leininger et al. 2021a, p. 3).

Based on this definition, social cohesion has three attributes: cooperation for the common good, trust and inclusive identity. All three attributes have a horizontal and a vertical dimension.

The first attribute is *cooperation for the common good*. When many people/groups cooperate for interests that go beyond—and sometimes even conflict with—those of the individuals involved it is a clear sign of high social cohesion because people who cooperate for the common good do care about society. Cooperation among individuals and groups represents that horizontal dimension, while cooperation between individuals/groups and state institutions represents the vertical dimension (Chan et al. 2006). For instance, the *maed magarat* (“dish sharing”) in Ethiopia—a food-sharing initiative between neighbours to counter the effects of the first wave of the Covid-19 pandemic (Leininger et al. 2021b, box 4)—is a form of horizontal cooperation. In turn, investing time to take part in participatory budget processes to define the purposes of public spending is an example of vertical cooperation for the common good.

The second attribute is *trust* (Chan et al. 2006; Dragolov et al. 2013; Langer et al. 2017; Schiefer and van der Noll 2017). Social cohesion includes two types of trust: generalised trust and institutional trust (Fukuyama 2001; Zerfu et al. 2009; Langer et al. 2017). Generalised trust is the “ability to trust people outside one’s familiar or kinship circles” (Mattes and Moreno 2018, p. 1) and it captures the horizontal dimension of social cohesion. Institutional trust, instead, refers to the trust towards the core, structural public institutions of a country (Mattes and Moreno 2018), and thus covers the vertical dimension.

The third attribute of social cohesion is *inclusive identity*. Most people feel they belong to different groups, and thus have several identities (such as religion, ethnicity, gender, village, family, class). A socially cohesive society is one in which individuals can have different identities and yet live together in a peaceful way, and where a minority with a shared identity does not dominate the majority with a collective identity. In other words, different group identities tolerate, recognise and protect each other while state institutions support such tolerance for different identities. In cohesive societies, individuals can still have different group identities but they should also have a feeling of mutual belonging to a broader unity (the nation) that is more than the sum of its members and can bridge identities.

There are still diverging views in the literature, especially regarding some potential ingredients of social cohesion. As stressed in a comprehensive review article by Schiefer and van der Noll (2017), these ingredients would be “quality of life/well-being” and “inequality”. We do not integrate well-being and inequality into our concept of social cohesion for three reasons. First, in line with other scholars (Dragolov et al. 2013; Schiefer and van der Noll 2017; Burchi et al. forthcoming), we argue that social cohesion is a “macro-level” or “meso-level” phenomenon. It is thus a specific trait of a community, a country, a region or the world as a whole. The literature on well-being, instead, focuses on individuals or households as units of analysis and refers to their living conditions in different life domains (Sen 1985). Second, it is problematic to include inequality as one of the constitutive elements of social cohesion as a notable number of studies do (Langer et al. 2017; Canadian Council on Social Development 2000; Berger-Schmitt 2000). It would imply that, by construction, societies that are more unequal are less socially cohesive. While it is plausible to expect a (negative) relationship between inequality and social cohesion, incorporating the former in the definition of the latter does not allow for testing it empirically. Third, in view of the objectives of this SI, having well-being or inequality as integral parts of the concept of social cohesion would generate particular problems. The expansion of well-being and the reduction of inequality are often considered two direct objectives of social protection. If included in the concept of social cohesion, social cohesion would be identified as a primary goal of social protection, as well. In other words, any policy that enlarges well-being or reduces disparities would automatically increase social cohesion: it is, instead, important to verify whether it contributes also to social cohesion through either of these two channels (or others).

## Conceptual Framework: Relationship Between Social Protection and Social Cohesion

**Fig. 1:**
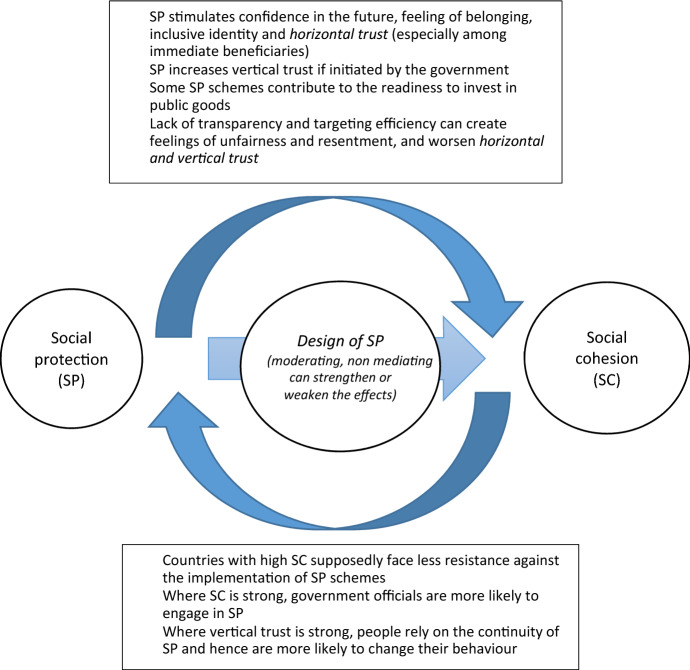
Main mechanisms between social protection and social cohesion. *Source* Authors

There are good conceptual arguments for the assumption that social protection and social cohesion affect each other (see Fig. 1).

As highlighted in the second section of this article, the goal of social protection is to reduce poverty and vulnerability and to contribute to pro-poor growth (Molyneux et al. 2016). In this SI, we argue that social protection can contribute to societal and political development, as well. More concretely, we state that social protection *can* have positive effects on all elements of social cohesion: inclusive identity, trust and cooperation for the common good.

Households that are well protected against the most serious of their individual risks can be assumed to have more confidence in themselves, feel better included in society because they have more opportunities than other households do and, hence, feel less alienated from other groups of society (Babajanian et al. 2014). This includes the positive impact of effective measures to mitigate climate change. In addition, social protection is an important tool to reduce inequality, i.e. disparities between different parts of the population. It would thus contribute not only to the *inclusive identity* (feeling of belonging) of beneficiaries but also to their *trust* in other members of society (*horizontal trust)*—even if these belong to other segments/groups of society (upper arrow in Fig. 1).

These effects can be particularly strong if social protection schemes incentivize interactions between members of different societal groups (such as in the case of CfW schemes where people with different origins work side by side for the common good, including females and males). Their cooperation in the creation of a common good can foster the acceptance into a group of individuals from outside that group, and the acceptance and legitimacy of social protection schemes in a society.

In addition, we assume social protection schemes strengthen *vertical trust*—at least if they are implemented or financed by the state (Burchi et al. 2020; Haider and Mcloughlin 2016; Loewe and Zintl 2021). Their beneficiaries are likely to be grateful to the actors that support them financially or by providing efficient instruments to deal with risk and poverty.^1^ Consequently, the overall trust of beneficiary households in public institutions is likely to increase—at least if social protection schemes are universal or well targeted at those in need (see below). Social protection thereby establishes a stronger relation between citizens and the government (Babajanian 2012). As a result, citizens tend to be more willing to accept their current government and the given political order, and to invest in public goods such as public order, the tidiness of streets or communal action (Burchi et al. 2020; Loewe et al. 2020). This effect on vertical trust is found to be especially relevant in climate change mitigation policies, where both cash transfers and trust in government play a key role (Klenert et al. 2018).

The intensity of all these effects depends on the design and implementation of social protection schemes (middle arrow in Fig. 1). For example, trust in the government is likely to increase mainly if the social protection schemes are set up or are effectively financed by the government and if the population is aware of this fact. If, however, social protection schemes are run and financed by non-governmental organisations or foreign donors, they might even have negative effects on citizens’ trust in their state.

Good communication can also be helpful. Vertical trust and cooperation is likely to increase more if the government gives a clear explanation of the rationale for the existence and design of a social protection scheme and that it is financed by scarce public resources. The most effective strategy to foster trust in the government is to establish social protection as a citizens’ right rather than as poverty relief (Evans et al. 2019; Vidican Auktor and Loewe 2021).

In addition, social protection’s effect on vertical trust is likely to be stronger if membership and targeting criteria are reasonable and transparent, and if citizens have reason to believe that the targeting is rule-based and fair in practice. In contrast, we can assume that high errors of inclusion and exclusion (and even rumours about it) have negative effects on citizens’ trust in the government. Lack of transparency can create feelings of unfairness and resentment as well, thereby worsening horizontal trust (Molyneux et al. 2016). In particular, it can create conflicts between direct programme beneficiaries and those excluded but perceived to be in similar conditions (Adato 2000; Adato and Roopnaraine 2004; Loewe et al. 2020). Even worse could be a situation whereby these programmes are targeted based on political considerations, or are at least perceived as such by the population. For example, social protection programmes often benefit mainly the middle class rather than the poor (Loewe 2009a), which can be intentional or not but in any case intensifies existing inequalities and hence weakens both horizontal and vertical trust (Köhler 2021). Moreover, some schemes, such as cash transfers targeted at the poor, can increase stigma and thus reduce social inclusion and social cohesion when not adequately designed (Li and Walker 2017; Loewe et al. 2020; Roelen 2017). Finally, if these programmes are not endorsed by the sections of society not directly addressed by these interventions, the net effect may be negative. In the best case, the target population itself participates in the design and implementation of social protection programmes, which adds to the positive effects on horizontal and vertical trust (Sabates-Wheeler et al. 2020; Loewe et al. 2020).

Likewise, the effect of social protection on people’s inclusive identity rises with the level and reliability of benefits. And the way beneficiaries are treated by government officials certainly plays a role as well (middle arrow in Fig. 1). Also, social protection supposedly improves the vertical dimension of all attributes, assuming they are universal rather than differentiated according to social groups (such as employment-related social insurance schemes, programmes of professional or trade unions or geographically targeted social transfer schemes). Poverty-targeted programmes may also be acceptable to large parts of the population if their targeting criteria are just, transparent and easy to understand. In any case, social transfer schemes are likely to generate stronger effects on social cohesion than social insurance schemes, whereby members finance their own benefits.

Though this SI mostly focuses on the effect of social protection on social cohesion, it also touches on the reverse relationship. In addition to being a goal in itself, social cohesion is also crucial for the implementation, design and effectiveness of social protection schemes (lower arrow in Fig. 1).

First, policy-making depends highly on the readiness of policy-makers to set up social protection schemes benefitting not just their clientele or peer group (ethnicity) but the entire population, or poor and other vulnerable groups in particular. Supposedly, this readiness is higher in countries with strong horizontal and vertical trust and cooperation for the common good. In addition, governments of countries with high social cohesion are less likely to face resistance against the implementation of social protection schemes for the poor and vulnerable. Recent studies in the context of Covid-19 have highlighted that trust in government is crucial for the selection of, and compliance with, containment policies (Bargain and Aminjonov 2020; Devine et al. 2020). This shows that high social cohesion enhances governments with public confidence. When social cohesion is weak, however, it may be much more difficult for governments to set up social protection schemes successfully, as citizens may feel resentful about social protection programmes they do not like, which may ultimately foster grievances in society (Abrams et al. 2020; Wilkinson et al. 2017).

Second, policy implementation benefits from social cohesion, too. Where social cohesion is strong, government officials are more likely to take actions that enhance the welfare of the population as a whole, and less likely to neglect their duties, misappropriate public funds and give preferential treatment to their peer group (family, home province, friends etc.). As a result, social protection schemes are more efficient and functional.

Third, policy reception is possibly even more important. Where vertical trust in the government is strong, people rely on the continuity of social protection policies and schemes and hence are more likely to change their behaviour (e.g. invest savings rather than hoarding them because they feel safe). Where sense of belonging and horizontal trust are strong, beneficiaries are more likely to share their benefits with other households and invest in social capital. As a result, the effectiveness of social protection schemes increases, including their multiplier effects. For traditional social protection schemes (solidarity networks among neighbours and friends) based on societal structures, social cohesion is even more important. They cannot work without horizontal trust, and if horizontal trust and sense of belonging are particularly strong, traditional social protection schemes are even based on generalised, rather than balanced, reciprocity, i.e. they function like an insurance rather than a mutual credit club. Where people have lived together for most of their lives and hence can trust each other, they are ready to help relatives, neighbours and friends in need without an expectation that their support will ever be paid back. Everybody receives support from those who are able to provide it, and gives support whenever they can to whatever person is in need—but this other person is not necessarily the same person that has provided help before. The reciprocity is thus between individuals and the community rather than between individuals, such as in the case of balanced reciprocity (Cronk et al. 2019; Loewe 2009b).

In this SI, we would have liked to discuss equally both directions of the relationship between social protection and social cohesion. Much of the discussion in subsequent sections focuses, however, on the effects of social protection on social cohesion. The reasons are two-fold: the increasing importance of social cohesion as a policy outcome and goal, which also feeds into empirical research agendas, and the fact that there is even less empirical literature on the effects of social cohesion on social protection than on the effects of social protection on social cohesion. This is possibly for two reasons. First, testing the effects of social cohesion on social protection requires variance in social cohesion, which exists mainly in cross-country comparisons, which are often impossible because of the lack of comparable data on social protection systems. Second, given the presence of several confounding factors, it is difficult to attribute differences in social protection programmes clearly to differences in the level of social cohesion.

## Empirical Evidence

Unfortunately, empirical evidence for the assumed bi-directional relationship between social protection and social cohesion is limited and scattered because social cohesion is hardly ever an explicit goal of social protection programmes and hence rarely considered in monitoring and evaluation reports. The few existing empirical studies define social cohesion in quite different ways, but most of them operate with attributes that are no different from, or very similar to, the ones we use: cooperation for the common good, trust and inclusive identity. Unfortunately, the bulk of the studies focus on the horizontal dimension of these three attributes. In addition, studies in this SI applied different research methods, which do justice to their specific research subjects and questions.

A number of studies are providing empirical evidence of the positive effects on the horizontal dimension of social cohesion. Most of them look at cash transfer schemes in sub-Saharan Africa or Latin America. Adato (2000), for example, conducted focus group discussions with different actors in 70 communities across six states of Mexico and finds that cash transfers have positive effects on horizontal trust. Two studies using both survey and experimental data document that a conditional cash transfer in Colombia has increased beneficiaries’ willingness to cooperate with each other (Attanasio et al. 2009, 2015). Relying on existing secondary data, primary data and the implementation of different qualitative and participatory methods in Yemen, West Bank and Gaza, Kenya, Uganda and Mozambique, Pavanello et al. (2016) confirm that both social insurance and assistance schemes can contribute to horizontal trust and inclusive identity by promoting local economic development. FAO (2014) provides evidence from several cash transfer schemes in sub-Saharan Africa indicating their positive impacts on social relations and participation in community events. ORIMA and the Asia Foundation (2020) argue that Timor-Leste’s Covid-19 cash transfer programme has a positive effect on horizontal trust: 82% of the population stated that Covid-19 had brought their community together, in contrast to 70% immediately before the pandemic. Also in this SI, Beierl and Dodlova (2022) find that CfW activities in Malawi increase the readiness of people to invest in public goods as well as to interact with others from the same or a different societal group. Andrews and Kryeziu (2013) provide evidence that CfW programmes in Ethiopia and Yemen have improved social cohesion by citizen participation in programme design. Roxin et al. (2020) find that CfW schemes in Turkey and Jordan have contributed to horizontal trust and the sense of belonging of participants and non-participants. Zintl and Loewe (2022, in this SI) confirm the finding for Jordan. UNHCR (2019) also finds that the Kalobeyei Integrated Social and Economic Development Programme (KISEDP) in Kenya, which enables refugees to purchase supplies from local shops and thereby promote interactions, has positive effects on horizontal trust between refugees and locals in Turkana. Likewise, Köhler (2021) presents some case studies to show that social protection programmes reduce poverty and thereby contribute to social inclusion, the overall satisfaction of people and, ultimately, social cohesion. Reeg (2017) suggests that the existence of social protection programmes raises the opportunity costs of being part of an armed group. And two studies (Lehmann and Masterson 2014; Valli et al. 2019) have assessed quantitatively the impacts of specific social protection programmes in refugee settings, providing initial evidence of positive effects on social relations among refugees and between refugees, and in one case also between refuges and local communities.

However, few studies suggest negative effects of social protection programmes on social relations (Adato and Roopnaraine 2004; Cameron et al. 2013; Kardan et al. 2010; Pavanello et al. 2016). In the majority of cases, the authors find that the lack of transparency and/or clarity in the targeting of beneficiaries generated feelings of jealousy among households that did not benefit from the programmes, thus increasing tensions with beneficiaries (Molyneux et al. 2016; Roelen 2017; Sumarto 2020; Burchi and Roscioli 2022 in this SI; Camacho 2014 in this SI). In addition, benefitting from social protection can also bring stigma and lower social cohesion (Hochfeld and Plagerson 2011). In a study based on individual interviews with different actors in Sri Lanka, Godamunne (2016) shows that disrespectful treatment by government officials and delays in the transfer of benefits weaken the vertical trust of beneficiaries of a social transfer programme. Roelen et al. (2022, in this SI) provide insights into different graduation programmes in Burundi and Haiti, showing that social protection can have positive and negative effects at the same time. While it can contribute to dignity, participation in social activities and sense of belonging, stringent targeting and discretionary provision of benefits can, in time, undermine trust among non-participants.

The evidence concerning the relationship between social protection and the vertical dimension of social cohesion is even scarcer and hence even less conclusive. Building on experimental data, Evans et al. (2019) find that a conditional cash transfer in Tanzania significantly increased vertical trust in local leaders and a self-reported willingness to participate in local projects. And this effect seems to be higher when beneficiaries are better informed about the central role played by the local government. In Brazil, however, Bolsa Familia did not reach the same positive results because beneficiaries did not believe that the designed institutional space to ensure their representation—the municipal-level councils—were “truly available to them for participation, monitoring, and accountability” (Molyneux et al. 2016, p. 1093). Other studies find negative effects of social protection schemes on societal perceptions of government (Aytaç 2014; Bruhn 1996; Guo 2009). Likewise, Zepeda and Alarcón (2010) show that social protection programmes foster vertical trust only if they are institutionally sustainable. Gehrke and Hartwig (2018) conduct an extensive literature review on public work programmes and finally suggest that the involvement of foreign donors in social protection policies can harm vertical trust. Zintl and Loewe (2022, in this SI) provide evidence in support of this assumption; they find CfW programmes to have positive effects on horizontal trust in Jordan; however, they report also that these same effects are much weaker where participants are aware of the fact that foreign donors rather than the national government have set up the respective CfW schemes. In addition, the effect is also much weaker if the targeting of transfers is perceived as unfair or non-transparent. Similarly, Camacho (2014) finds that the conditional cash transfer in Peru increases vertical trust only among the beneficiaries, and decreases it among non-beneficiaries. Köhler (2021) presents anecdotal evidence that the introduction of a social pension and a child benefit scheme in Nepal has been a major factor in the increase in vertical trust in Nepal after 2009, while the dismantling of pension schemes in Chile led to a decrease in vertical trust.

Looking at the reverse side of the relationship, we find even less empirical evidence. Based on qualitative analysis, Hossain et al. (2012) find that the Indonesian unconditional cash transfer programme *Bantuan Langsung Tunai* had positive effects on different outcomes only in high social cohesion communities. In a study covering four Asian countries, Babajanian et al. (2014) find that the impacts of social protection schemes depend substantially on the local institutional setting and, above all, on the nature of the relationship among social groups. Indeed, where gender and ethnic disparities were high especially due to existence of discriminatory rules against women and specific groups, programme performance was lower. Roelen et al. (2022, in this SI) shows that the quality of horizontal relationships at the community level plays an important role in the success of two different graduation programmes in Haiti and Burundi.

## Findings of this SI and Their Implications for Future Research

The remainder of the articles in this SI contribute to filling some of the gaps outlined in this introduction in two ways. The first is by using a common understanding and definition of social cohesion. Some papers focus on just some components of the definition (especially trust), but they all share a common understanding. This facilitates a comparison of the findings across papers. Second, the articles look at several mechanisms linking social protection and social cohesion, and also represent a good balance between qualitative and quantitative methods. In addition, the aforementioned comparability advantage of the SI is strengthened by the different contexts and countries considered, as well as the different social protection programmes analysed, ranging from long-term to short-term ones, from conditional and unconditional cash transfers and public works schemes to graduation (or social protection plus) programmes^2^ and contributory social insurance schemes. Table 1 provides a brief overview of the main features of the different articles of the SI.

**Table 1:** Articles in this SI of EJDR on social protection and social cohesion

Author(s)	Geographic focus	Social protection scheme(s)	Attribute(s) and dimension(s) of social cohesion	Method
Burchi, Loewe, Malerba and Leininger	Global	All	All attributes and dimensions	Literature review and conceptual considerations
Roelen, Kim and Leon-Himmelstine	Burundi and Haiti	Graduation programmes	Mainly cooperation for the common good and inclusive identity on two dimensions Plus effects of social cohesion on social protection	Qualitative analysis of semi-structured discussions, interactive activities and focus group discussions
Burchi and Roscioli	Malawi	Graduation programmes	All attributes and dimensions	Mixed-method design: experimental design and primary household data; econometric analysis; focus group discussions and individual interviews
Beierl and Dodlova	Malawi	Cash for work programmes	Cooperation for the common good	Quantitative analysis of three household surveys
Zintl and Loewe	Jordan	Cash for work programmes	All attributes and dimensions	Quantitative analysis of census data and qualitative content analysis of key informant interviews
Strupat	Kenya	All existing contributory and non-contributory social protection schemes	All attributes and dimensions	Quantitative analysis of representative household survey data
Ongowo	Kenya	Social services	Mainly inclusive identity and horizontal trust	Qualitative content analysis of key informant interviews
Malerba	Global	Social protection policies used in combination with climate mitigation policies	All attributes and dimensions	Literature review and econometric analysis

The majority of the articles advance our understanding of the effects of social protection on social cohesion. *Burchi and Roscioli*, for example, look at the effects of an integrated social protection programme on social cohesion in Malawi using a mixed-methods approach. Specifically, they exploit an experimental design and primary household data for about 800 households in total to investigate the impact of three different components of the programme on a set of indicators for the trust and cooperation attributes. Informed by the results of the econometric analysis, they then examine the contribution of one specific component—participation in the saving groups—through focus group discussions and individual interviews. The study shows no concrete effect of a lump-sum payment on social cohesion, but a positive effect of both the training and participation in savings groups on within-group trust and (economic and non-economic) cooperation. Conversely, vertical trust towards local institutions and horizontal trust towards other village members declined, in particular due to jealousy and tensions arising from the targeting of social protection. The authors thus underline the possible limitations of just giving cash, as well as the potential of savings groups.

Still in Malawi, *Beierl and Dodlova* investigate whether a public works programme effects cooperation for the common good. The authors address this research question through quantitative analysis applied to primary and secondary data. The primary data, collected in two waves (2017 and 2019), cover 500 randomly selected households; secondary data are from the nationally representative integrated household survey conducted by the World Bank in three waves (2010, 2013, and 2016). The paper finds that the scheme improves cooperation among community members and speculates that this may, in turn, improve trust among community members and the perception of state institutions.

*Strupat* examines the effects of social protection on social cohesion during a large covariate shock such as the Covid-19 pandemic in Kenya. He does so econometrically, by using a difference-in-difference model and household data collected before and after the Covid-19 pandemic. His analysis suggests that social assistance has no statistically significant preserving effect on social cohesion overall.

*Ongowo* presents the results of qualitative research on the effects of social protection on social cohesion, focusing on street children in Kenya. The author conducted comprehensive qualitative content analysis of key informant interviews with twelve government officials, and in-depth qualitative interviews with twelve randomly selected former street children who previously benefited from social protection programmes. He finds that social protection can be an important tool to build social capital and solidarity. In particular, he concludes that social protection programmes improve the chances of street children developing a career, reduce public resentment towards street children and, thus, enhance various aspects of social cohesion.

*Zintl and Loewe* in turn look at social cohesion in the context of state fragility and migration, with a focus on donor-funded programmes. They analyse the effects of public works/CfW programmes in Jordan on participants and non-participants, in both cases Syrian refugees and Jordanian locals, females and males. Their results are based on qualitative analysis of key informant interviews (281 with CfW participants and non-participants at nine CfW sites all over Jordan, 99 with neutral observers at local and national levels), four group discussions and quantitative analysis of a census among all participants of one specific CfW programme. The results confirm effects on the sense of belonging and horizontal trust of participants and non-participants, refugees and locals. They provide evidence in particular for a positive effect on women being more active in the economy and the society. The results for vertical trust, however, are more ambiguous because many Syrians and Jordanians attribute positive effects to donor support rather than to Jordanian authorities.

Other papers in the SI look at the broader picture by also including the effects running from social cohesion to social protection. *Roelen *et al*.* conduct extensive qualitative analysis to investigate the bi-directional relationship between social protection and social cohesion in Burundi and Haiti. In particular, key informant interviews and focus group discussions were performed with programme participants (male and female) and programme staff. Data collection was based on semi-structured discussions as well as interactive activities such as ranking exercises. They find that the existing programmes have strengthened some aspects of social cohesion, such as dignity and positive identity, whilst also having negative effects on others, such as sense of belonging and togetherness. However, they also find that social cohesion enhanced the positive effects of social protection programmes.

*Malerba* looks at social protection and social cohesion in the context of climate change mitigation. This is important, as climate mitigation policies are strictly related to socio-economic development in low- and middle-income countries. While some of the issues have been investigated in separate literatures, there was a lack of unifying framework or empirical analysis when considering the combined effects of social protection and social cohesion on the implementation of climate mitigation. In more detail, the econometric analysis employs data collected in 34 countries (24 high-income and 10 lower income) in a multilevel model framework. The data used collected preferences for environmental policies as well as other relevant information. The results show that social cohesion in the form of trust is positively correlated with support for climate mitigation. Conversely, social protection has positive effects only in high-income countries but not in middle-income countries; this suggests low complementarity between climate and social policies and higher prioritisation of social goals in lower-income contexts.

In sum, the papers in this volume provide support and empirical evidence to different aspects of the relationship between social protection and social cohesion, as outlined in the conceptual framework. However, despite the important contribution that the SI makes to the topic, further research needs to be done on the remaining critical gaps. One of these is the impact of social cohesion on the effectiveness of social protection, as this SI focuses more on the inverse relationship. Such research can definitely benefit from applying the definitions of social protection and social cohesion used by all authors of the articles in this SI.

Future research should also address the other gaps outlined in the first section. Social cohesion has become prominent only in recent years (which affects availability of data collection and programme evaluation in the context of social protection programmes) and the relationship between social protection and social cohesion is not direct and straightforward. These facts make the empirical analysis challenging from a methodological point of view. Therefore, better data, which will hopefully become increasingly available, can improve the empirical evidence and the knowledge of these issues.

And as a third research gap, it remains to be seen how the ongoing expansion of social protection programmes in the aftermath of the COVID-19 pandemic (Gentilini et al. 2020) can be better linked with the goal of improving social cohesion.

## Footnotes

1 Here, we do not refer to the incumbent governments only. Recipients of social protection are likely to engage and eventually appreciate just as much or even more so the work of local administrators and officials in social protection schemes. Moreover, in the ideal scenario, programmes like cash transfers are long-standing programmes, so that improvements in citizen–state relationships go well beyond those of the simple citizen–incumbent government.

2 Graduation programmes are multi-component programmes that integrate traditional social protection instruments, such as cash or food transfers, with additional services, such as various forms of training and mentoring. Their objective is to ensure a sustainable graduation out of poverty for its beneficiaries.
